# Notch signaling regulates remodeling and vessel diameter in the extraembryonic yolk sac

**DOI:** 10.1186/1471-213X-11-12

**Published:** 2011-02-25

**Authors:** Jessica N Copeland, Yi Feng, Naveen K Neradugomma, Patrick E Fields, Jay L Vivian

**Affiliations:** 1 1Department of Pathology and Laboratory Medicine and Institute for Reproductive Health and Regenerative Medicine, University of Kansas Medical Center, Kansas City, KS 66160, USA

## Abstract

**Background:**

The signaling cascades that direct the morphological differentiation of the vascular system during early embryogenesis are not well defined. Several signaling pathways, including Notch and VEGF signaling, are critical for the formation of the vasculature in the mouse. To further understand the role of Notch signaling during endothelial differentiation and the genes regulated by this pathway, both loss-of-function and gain-of-function approaches were analyzed in vivo.

**Results:**

Conditional transgenic models were used to expand and ablate Notch signaling in the early embryonic endothelium. Embryos with activated Notch1 signaling in the vasculature displayed a variety of defects, and died soon after E10.5. Most notably, the extraembryonic vasculature of the yolk sac displayed remodeling differentiation defects, with greatly enlarged lumens. These phenotypes were distinct from endothelial loss-of-function of RBPJ, a transcriptional regulator of Notch activity. Gene expression analysis of RNA isolated from the yolk sac endothelia of transgenic embryos indicated aberrant expression in a variety of genes in these models. In particular, a variety of secreted factors, including VEGF and TGF-β family members, displayed coordinate expression defects in the loss-of-function and gain-of-function models.

**Conclusions:**

Morphological analyses of the in vivo models confirm and expand the understanding of Notch signaling in directing endothelial development, specifically in the regulation of vessel diameter in the intra- and extraembryonic vasculature. Expression analysis of these in vivo models suggests that the vascular differentiation defects may be due to the regulation of key genes through the Notch-RBPJ signaling axis. A number of these genes regulated by Notch signaling encode secreted factors, suggesting that Notch signaling may mediate remodeling and vessel diameter in the extraembryonic yolk sac via autocrine and paracrine cell communication. We propose a role for Notch signaling in elaborating the microenvironment of the nascent arteriole, suggesting novel regulatory connections between Notch signaling and other signaling pathways during endothelial differentiation.

## Background

The formation of the vascular system is essential for nutrient and waste transport in the growing embryo. In mice, the developing vasculature initially forms in intraembryonic and extraembryonic regions. In the extraembryonic yolk sac at approximately E7.0-7.5, angioblasts are formed from the differentiation of mesodermal cells. These angioblasts differentiate into endothelial cells, elaborate cell contacts, and lumenize into simple tubes; resulting in the formation of a capillary plexus network [1,2]. The simple plexus of the yolk sac is remodeled and refined after E8.5 to form the larger diameter vessels. During this process, extensive movements of endothelial cells within the plexus occur through a process termed intussusceptive arborization [3], reallocating cells from the capillaries to larger vessels, to assemble a more complex vasculature network [4,5]. This process forms the vitelline arteriole and venule, which participate in the contiguous blood flow with the embryonic vasculature, concomitant with the initiation of flow after E9.0. Although likely context dependent, vessel remodeling also occurs in the adult, during wound healing, reproductive cycling, and tumor progression [6]. More work needs to be done to define the shared and distinct regulatory paths that control vascular differentiation in the various sites of development and in the adult.

Both vasculogenic and angiogenic processes are highly regulative, and under the control of a number of signaling pathways, including the vascular endothelial growth factor (VEGF) pathway, the Notch pathway, and the transforming growth factor-β (TGF-β) pathway, among others [7-10]. Notch signaling is an evolutionary conserved pathway and a determinant of cell fate [11]. Four Notch receptors (Notch 1-4) exist in mice and human along with five ligands (Jagged1 and -2, and Dll1, -3, and -4) [12]. The Notch receptors are activated upon ligand binding, which initiates the proteolysis of its intracellular domain (N-ICD). The N-ICD translocates to the nucleus where it interacts with a family of DNA-binding proteins, termed recombination signal-binding protein for immunoglobulin kappa J region (RBPJ; also known as C-promoter binding factor 1, CBF1), forming a transcriptional activator complex at the regulatory elements of target genes, thereby directing changes in gene expression transcription [12].

Much work has been done to define the roles of the Notch signaling pathway during vascular differentiation. *Notch1*, *Notch4*, *Dll4*, *Jagged1*, and *Jagged2 *are all expressed in the arterial endothelium of vertebrates, *Notch4 *being solely expressed in the endothelia of mouse embryos [13,14]. Mutations in these genes lead to defects in the vasculature, many of which are embryonic lethal. Mutant mice lacking *Notch1 *do not survive post E11.5 and harbor defects in vascular remodeling in the embryo, yolk sac, and placenta [15]. Deletion of *Notch4 *has no visible effect and embryos are viable; however, *Notch1^-/-^Notch4^-/- ^*double mutants have more severe vascular phenotypes than the *Notch1^-/- ^*and are embryonic lethal at E9.5 [10,16]. Expression of an activated form of *Notch4 *or *Notch1 *also leads to vascular defects similar to those seen in the *Notch1^-/- ^*and *Notch1^-/-^Notch4^-/- ^*mice, as well as embryonic lethality at ~E10 [17,18].

Although Notch clearly plays important roles in the formation of the early embryonic vasculature, very little is known about the nature of the downstream targets in vivo, and how changes in Notch activity elicit the observed morphological processes. In vitro analysis has indicated novel Notch targets, including receptors of the VEGF family, VEGFR-3 (*Flt4*) and VEGFR-1 (*Flt1*) [19,20]. Given that several signaling cascades are required for the morphological differentiation of the embryonic vasculature, it is likely that these pathways interact during vascular development.

To better define the activity of Notch signaling in vascular differentiation, a detailed morphological and molecular analysis was performed using developmental models in which the Notch signaling pathway is altered. A gain-of-function Notch1 transgenic model showed that expanded Notch1 signaling in the early vasculature results in defects in embryo growth, defective differentiation during remodeling of the yolk sac vasculature, altered patterns of gene expression, and ultimately embryonic lethality. These phenotypes were compared to embryos lacking Notch signaling in the endothelia, via a tissue-specific loss of RBPJ function. Embryos lacking endothelial *Rbpj *exhibited distinct growth, vascular, and gene expression defects compared to the Notch1 gain-of-function model. Gene expression analysis in the yolk sacs of these models demonstrated altered patterns of expression of a distinct subset of Notch targets. Additionally, several secreted ligands, including the TGF-β ligand TGFβ2 and the VEGF ligands VEGFC and Placenta Growth Factor (PlGF), were altered in these models, suggesting a role for an altered VEGF signaling pathway in the observed phenotypes of these models. Our data suggest a model in which Notch signaling in the endothelia is critical for elaborating a specialized local environment of the developing arterial vasculature, by influencing the expression of secreted factors, which may be important in autocrine or paracrine signaling to direct further morphological differentiation of the vasculature during remodeling.

## Results

### Conditional transgenesis to modulate Notch signaling in the early endothelia

**Figure 1:**
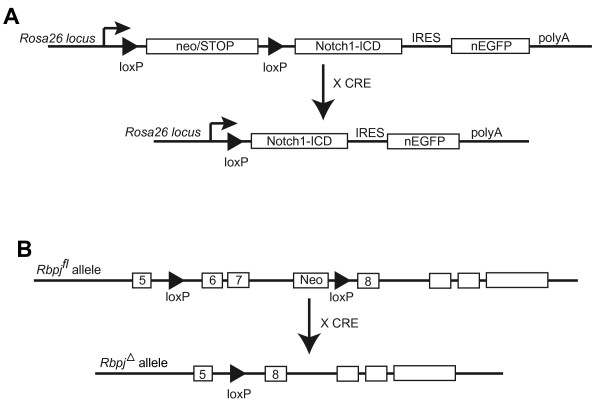
**Conditional mouse EC-N1ICD and EC-Rbpj-KO transgene constructs**. (A) Upon crossing of mice that carry the *Rosa^Notch ^*transgene to a transgenic mouse that expresses CRE in the endothelia, recombination removes the neomycin cassette and induces expression of the *Notch1 *intracellular domain only in endothelial cells. (B) Upon crossing of mice that carry the *Rbpj^f ^*transgene [22] to a transgenic mouse that expresses Cre in the endothelia, recombination will remove the neomycin cassette along with exons 6 and 7, which encode for the DNA binding domains of RBPJ, abrogating the promoter activity of RBPJ only in endothelial cells.

To further understand the functions of Notch signaling in early vascular development, genetic models were employed to modulate Notch activity in the embryonic endothelia. These models employ endothelial-specific Cre-mediated recombination in vivo. To activate and expand Notch1 signaling in the endothelia, a transgenic line *Rosa^Notch ^*[21] was used, which harbors a NOTCH 1 intracellular domain (N1ICD) cDNA downstream of a floxed STOP fragment targeted to the *Rosa26 *locus. Removal of the STOP cassette through *loxP*-mediated recombination, via an endothelial CRE expressing transgene, results in expression of N1ICD in endothelial cells (designated as EC-N1ICD embryos; Figure 1A). To delete Notch signaling in the early endothelia, a mouse line was used which harbors a conditional allele of *Rbpj *(*Rbpj^f ^*mice) [22]. Crossing these mice to endothelial-specific CRE transgenic mice results in the deletion of exons 6 and 7, which encode the DNA binding domain of *Rbpj*, abrogating the activity of RBPJ only in endothelial cells (designated as EC-Rbpj-KO embryos; Figure 1B). The conditional deletion would be predicted to disrupt both Notch1 and Notch4 signaling, which have known redundant functions in early embryonic vascular differentiation [10]. Ablation of *Rbpj *in the endothelia was used to assure a complete disruption of Notch signaling in the developing vasculature.

*Tie2-Cre *[23] or *Flk1-Cre *[24] transgenic mice were crossed to either *Rosa^Notch ^*or *Rbpj^f ^*mice. Each of these transgenes expresses the *Cre *recombinase gene and direct expression principally to the vascular endothelium. Identical phenotypes were observed when the *Flk1-Cre *and *Tie2-Cre *transgenic lines were crossed to the *Rosa^Notch ^*and *Rbpj^f ^*models (data not shown). Previous work has demonstrated that these Cre expressing transgenes exhibit restricted endothelial expression of Cre recombinase activity to the endothelial and hematopoietic lineages [24]. The early embryonic expression and recombinase activity of the *Tie2-Cre *and *Flk1-Cre *transgenes was confirmed by crossing these transgenes to a conditional *LacZ *reporter [25]. Both transgenes showed specific expression within the early endothelial and hematopoietic lineages at E8.5 (Additional File 1).

### Regulated Notch signaling is essential for the growth and development of the early embryo

**Figure 2:**
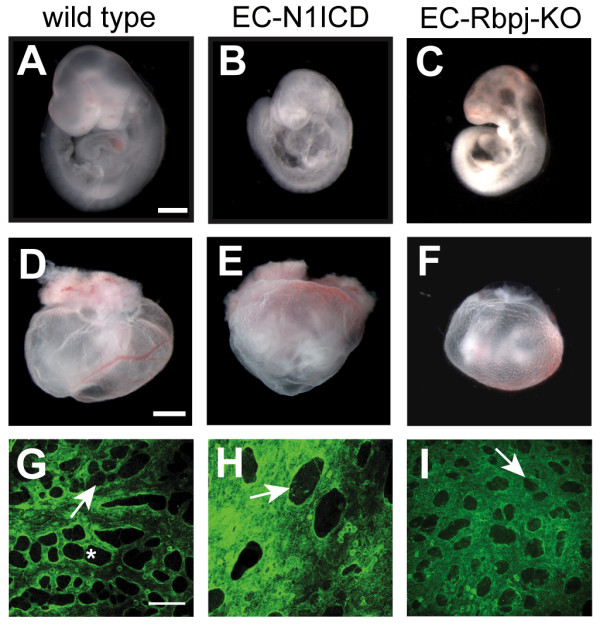
**Defects in growth and yolk sac vasculature remodeling in EC-N1ICD and EC-Rbpj-KO embryos**. (A-C) Lateral view of E9.5 wild type (A), EC-N1ICD (B), and EC-Rbpj-KO (C) embryos. EC-N1ICD and EC-Rbpj-KO embryos were smaller in size than the wild type and exhibited cardiovascular defects. (D-F) Whole mount E9.5 wild type (D), EC-N1ICD (E), and EC-Rbpj-KO (F) embryos with surrounding yolk sac. The EC-N1ICD and EC-Rbpj-KO yolk sac lacked the large, well-defined blood vessels seen in the wild type. The blood in the EC-N1ICD yolk sac collected near the attachment to the placenta. (G-I) Immunofluorescence image of E9.5 wild type (G), EC-N1ICD (H) and EC-Rpbj-KO (H) yolk sac visualized with an antibody to PECAM1. Wild type embryos showed a remodeled yolk sac vasculature, with both large and small caliber vessels. The EC-N1ICD yolk sac did not exhibit branching; all vessels were of a large caliber. The EC-Rbpj-KO yolk sac also failed to show vascular remodeling, although all vessels were of a small caliber. Scale bars are 500 μm (A-C), 1 mm (D-F) and 100 μm (G-I). Arrows (G-I), avascular inter-vessel space. Asterisk (G), capillary collapse.

To activate Notch1 signaling throughout the embryonic endothelia, female mice heterozygous for the *Rosa^Notch ^*transgene were crossed with male mice hemizygous for the *Tie2-Cre *transgene, and the resulting embryos were analyzed. At E8.5 the EC-N1ICD mice were morphologically normal and identical to the wild type siblings, with open neural folds and vascularized allantois characteristic of this time point (Additional File 2). Compared to stage-matched wild type embryos at E9.5, EC-N1ICD embryos exhibited an enlarged heart and a reduction in overall size (Figure 2A,B). Growth defects were much more pronounced at E10.5 (data not shown), and no viable embryos were observed after E10.5.

To ablate Notch signaling in the embryo, *Tie2-Cre *mice were used in a two-generation cross to generate *Tie2-Cre*; *Rbpj^f/f ^*embryos (EC-Rbpj-KO), which lack RBPJ binding activity in the endothelia. The EC-Rbpj-KO embryos displayed severe growth retardation defects at E9.5 similar to those observed in the EC-N1ICD embryos (Figure 2C). The morphological analyses of the gain-of-function and loss-of-function embryos were consistent with other models [10,17,18,26] and confirmed that the appropriate Notch signaling in the endothelia of the early embryo is critical for proper growth and development of the embryo.

### Vascular defects in EC-N1ICD and EC-Rbpj-KO embryos

A detailed comparison of the vasculature was performed to define the vascular defects in the embryonic and extraembryonic vasculature of EC-N1ICD and EC-Rbpj-KO embryos. At E8.5, no overt defects were observed in the developing vasculature of either the EC-N1ICD or EC-Rbpj-KO. In particular, the vascular plexus of the EC-N1ICD yolk sac, visualized by histochemical staining of endothelial cells with an antibody to PECAM1 (CD31), appeared unaffected when compared to stage-matched wild type embryos (Additional File 2; data not shown). The most severe defects were seen in the yolk sac of the developing embryo beginning at approximately E9.5. Gross morphological examination of the vasculature of the yolk sac by whole mount light microscopy showed that at E9.5 the EC-N1ICD yolk sac was the same size as the stage-matched wild type control; however these embryos lacked large diameter vessels (Figure 2D,E), indicating a failure in appropriate blood vessel remodeling occurring at this time point [5]. The EC-N1ICD yolk sac vessels did contain blood cells, although the blood tended to pool near the proximal end of the yolk sac adjacent to the chorioallantoic plate (Figure 2E). EC-Rbpj-KO embryos also lacked vascular remodeling in the yolk sac, failing to form the large vitelline blood vessels [26] (Figure 2F).

Immunofluorescence of PECAM1 stained E9.5 yolk sac revealed the failure of this remodeling in both the EC-N1ICD and EC-Rbpj-KO embryos in greater detail. In the EC-N1ICD embryos, no distinction between large caliber vessels and capillaries was observed (Figure 2H); instead, the vasculature consisted of vessels with an enlarged surface area with greatly decreased avascular inter-vessel space compared to wild type controls, as assessed by lack of PECAM1 expression ('pillars'; Figure 2G,H, arrows). In contrast to the EC-N1ICD vessel defects, the EC-Rbpj-KO embryos exhibited a qualitatively different vessel phenotype in the yolk sac. Although EC-Rbpj-KO embryos also exhibited a lack of vascular remodeling, the yolk sac of these embryos displayed numerous small avascular intervessel spaces (Figure 2I, arrow). These results indicated that the yolk sac vasculature of the EC-Rbpj-KO embryos failed to form the large vitelline blood vessels, reminiscent of the simple vascular plexus seen at E8.5.

**Figure 3:**
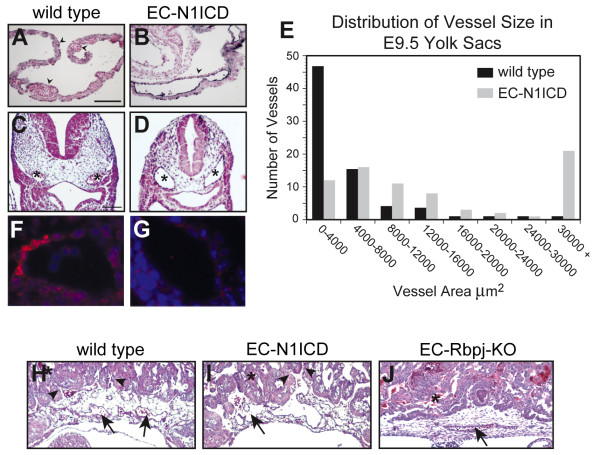
**Defects in vessel diameter in EC-N1ICD and EC-Rbpj-KO embryos**. Histological sections (lateral) of PECAM1 and NFR-stained E9.5 yolk sac (A, B) and embryos (C, D) at the level of the heart. The wild type yolk sac contained both large and small caliber vessels (A, arrowheads). The EC-N1ICD yolk sacs contained primarily large caliber vessels (B, arrowheads), however they were lumenized. The dorsal aortae of the EC-N1ICD embryos (D, asterisk) were approximately twice the area of wild type dorsal aortae (C, asterisk). (E) Distribution of vessel area in the yolk sac of wild type and EC-N1ICD embryos. Both wild type and EC-N1ICD embryos had an array of differently sized vessels. However, wild type yolk sac had a majority of vessels with an area of 0-4000 μm^2^, while EC-N1ICD contained many vessels with an area of 30000 μm^2 ^or greater. (F, G) Histological sections of E9.5 dorsal aorta stained with an antibody to smooth muscle α-actin. The dorsal aortae of the wild type contain SMA-positive cells (F), while the EC-N1ICD dorsal aortae are SMA-negative. (H-J) Histological sections of the placental vasculature in wild type (H), EC-N1ICD (I), and EC-Rbpj-KO (J) embryos. Blood vessels containing nucleated erythrocytes in EC-N1ICD placenta (I, arrow) were of larger caliber than blood vessels in wild type placenta (H, arrow). In both, the fetal vasculature invaded into the maternal portion of the placenta. The vessels of the EC-Rbpj-KO placenta (J, arrow) were small in size and had not invaded into the labyrinthine layer. Asterisk (H-J), maternal blood. Scale bars are 100 μm (A-D).

Histological sectioning of yolk sac tissue was performed to visualize the vessels in cross-section via PECAM1 immunochemistry and hematoxylin and eosin staining. In the wild type yolk sac a distribution of large and small caliber vessels were present and were filled with blood cells; in striking contrast, the EC-N1ICD yolk sac contained primarily large caliber lumenized vessels that contained blood cells (Figure 3A,B). Both wild type and EC-N1ICD embryos had a range of yolk sac vessel diameter. However, in wild type yolk sac a majority of the vessels measured consisted of small capillaries, while in the EC-N1ICD yolk sac a larger proportion of vessels consisted of a larger cross-sectional area. In EC-N1ICD yolk sac, approximately 36% of vessels measured had an area of 16000 μm^2 ^or greater, while in wild type yolk sac only 5% measured this size (Figure 3E). This enlarged vessel phenotype in response to Notch activation was observed in other sites of vessel differentiation in the developing embryo. The dorsal aortae (DA) of EC-N1ICD embryos were approximately twice the cross-sectional area of wild type embryos (Figure 3C,D, asterisks). The embryo-derived vasculature of the placenta of EC-N1ICD embryos did invade into the labyrinth layer of the placenta (Figure 3G, arrowheads), but exhibited greatly enlarged vessel diameter (Figure 3G, arrows). The EC-Rbpj-KO embryos exhibited a decrease in vessel diameter, including the DA, and arteriovenous malformations [26]. EC-Rbpj-KO embryonic-derived vasculature of the placenta was also reduced in size and did not invade the labyrinth layer of the placenta (Figure 3H, arrow). Immunostaining of E9.5 wild type and EC-N1ICD embryos using anti-SMA, indicated that the dorsal aortae of wild type are surrounded by smooth muscle cells, while the EC-N1ICD did not recruit any SMA-positive cells to the dorsal aorta [27] (Figure 3F,G).

The morphological analyses of the models of altered Notch signaling were consistent with previous analysis of Notch function in the vasculature [10,17,18,26]. These results point to multiple roles for Notch signaling in the formation of both the intra- and extraembryonic vasculature, including the remodeling of the yolk sac vasculature, the regulation of vessel diameter, and the invasion of the fetal vasculature into the labyrinth layer of the placenta.

### Identification of Notch regulated genes in EC-N1ICD yolk sac and EC-Rbpj-KO yolk sac

**Figure 4:**
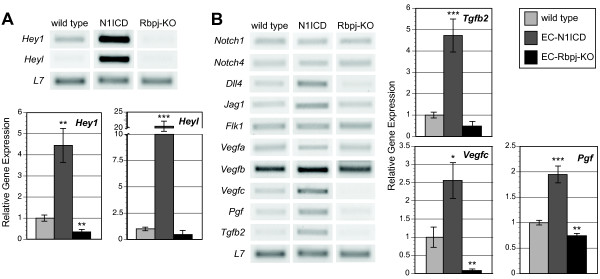
**Gene expression in transgenic EC-N1ICD and EC-Rbpj-KO embryo yolk sacs**. Semiquantitative RT-PCR analysis was used to confirm gene expression differences seen in whole genome microarrays. (A) RT-PCR analysis of *Hey1 *and *Heyl *in wild type, EC-N1ICD, and EC-Rbpj-KO E9.5 yolk sacs. Quantitated relative mRNA levels are shown in the lower graph. (B) RT-PCR analysis of genes critical for the formation of the early embryonic vasculature. Quantitated relative mRNA levels of select genes are shown in the graphs to the right. * p < .05, ** p < .01, *** p < .001 compared to wild type.

Gene expression defects in the Notch models were examined to determine potential molecular mechanisms of how altered Notch disrupts vascular differentiation, through altered expression of putative Notch targets. RNA from yolk sacs of E9.5 wild type, EC-N1ICD and EC-Rbpj-KO embryos (resulting from a cross with *Tie2-Cre *or *Flk1-Cre *mice) was isolated for gene expression analysis via microarray and semiquantitative RT-PCR. Whole genome microarray identified a large number of gene expression defects in these Notch models, many of which were confirmed with RT-PCR (Figure 4). To identify putative Notch targets, genes were categorized on the altered response in the gain-of-function and loss-of-function models (Additional File 3). Only a relatively small set of genes displayed upregulation in the EC-N1ICD model and downregulation in the EC-Rbpj-KO model, which would be suggestive of genes that are positively regulated by the Notch1-Rbpj axis. Interestingly, no genes were identified as being downregulated in the EC-N1ICD model and upregulated in EC-Rbpj-KO (Additional File 3).

The members of the Hairy/Enhancer of split-related (HES/HEY) family are well described Notch targets, many of which are directly regulated by Notch1 signaling via its binding to RBPJ [28] to regulatory regions of these loci. The microarray datasets revealed that in yolk sacs of EC-N1ICD embryos, both *Hey1 *and *Heyl *were highly induced, and expression of these genes was reduced in the EC-Rbpj-KO; these results were confirmed by semi-quantitative RT-PCR (Figure 4A). Expression of *Hey1 *and *Heyl *was increased 4.4-fold and 20.5-fold in the EC-N1ICD model respectively, and decreased to 35% and 46% of wild type levels in the EC-Rbpj-KO yolk sac tissue, respectively. Interestingly, the expression of other HES family members, such as *Hes1 *and *Hes6 *was not affected in these Notch models (data not shown), consistent with a context-dependent regulation of Notch targets [29,30]; i.e. Notch signaling invokes distinct downstream targets depending on both the cell type and the local environment.

The expression of the endogenous Notch ligands and receptors was also examined in detail. Previous work has suggested a potential autoregulatory loop for Notch signaling in the control of ligands and receptors [31,32]. Although expression of the Notch family receptors was not altered in either the EC-N1ICD or EC-Rbpj-KO yolk sacs, the Notch ligands Delta-like 4 (*Dll4*) and Jagged 1 (*Jag1*) showed higher expression in EC-N1ICD (2.5-fold and 2.0-fold, respectively) with little or no change in expression in EC-Rbpj-KO yolk sac. These findings point to specific Notch pathway targets in the extraembryonic yolk sac vasculature.

### Notch-Rbpj signaling regulates vascular expression of key signaling molecules

Given the defects in vessel diameter and remodeling in response to altered Notch activity, we wished to focus gene expression analysis on secreted factors. Genes encoding several known and putative secreted factors were differentially expressed in the Notch models (Additional File 4). Expression defects of a subset of these genes were confirmed via RT-PCR, with a focus on genes with putative roles in vascular differentiation and that display coordinate defects in expression in the EC-N1ICD and EC-Rbpj-KO models. Although the expression of some VEGF family members was not significantly different in yolk sac, the microarray datasets indicated that expression of the VEGF family members *Vegfc*, encoding VEGF-C, and *Pgf*, encoding Placenta Growth Factor (PlGF), were both increased in EC-N1ICD and *Vegfc *was decreased in EC-Rbpj-KO (Additional File 4). RT-PCR confirmed that *Vegfc *was upregulated 2.6-fold in the EC-N1ICD and decreased to 8% in the EC-Rbpj-KO compared to control yolk sac tissue; *Pgf *was upregulated 1.9-fold in the EC-N1ICD and decreased to 74% in the EC-Rbpj-KO compared to control yolk sac tissue. The secreted cytokine *Tgfb2 *also exhibited increased expression in the yolk sacs of EC-N1ICD yolk sac tissue (4.7-fold) and decreased expression in EC-Rbpj-KO (49% of wild type levels) (Figure 4B).

**Figure 5:**
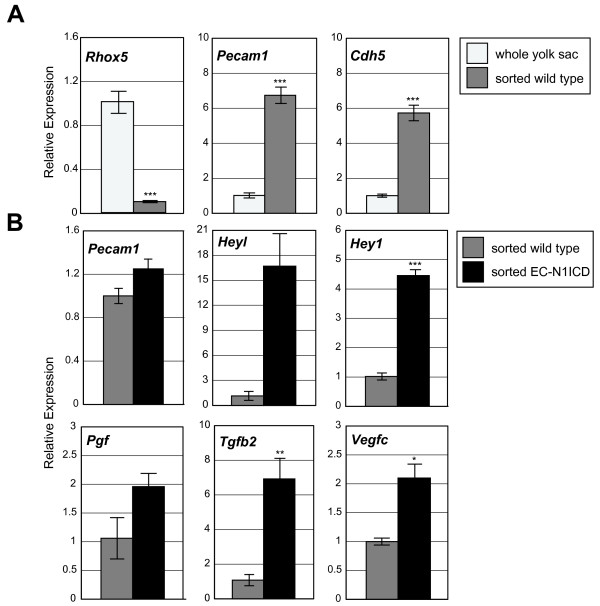
**Gene expression in transgenic EC-N1ICD PECAM1+ sorted yolk sac tissues**. Real time-PCR analysis was used to analyze gene expression differences seen in PECAM1+ sorted yolk sac cells. (A) Real time-PCR analysis of *Cdh5, Pecam1 *and *Rhox5 *in wild type E9.5 whole yolk sac tissues and sorted yolk sac endothelial cells. (B) Real time-PCR analysis of select Notch responsive genes. * p < .05, ** p < .01, *** p < .001 compared to wild type.

Given that the N1ICD was activated specifically in the endothelia in this transgenic model, we wished to confirm that the gene expression defects in this model are confined to the endothelial lineage, to determine if the gene expression defects are intrinsic to the endothelium, or are associated with another cell type within the yolk sac. To these ends, gene expression of Notch regulated genes was examined in endothelial cells purified via flow cytometry. Via fluorescent activated cell sorting (Additional File 5), PECAM1 + cells were isolated from dissociated yolk sacs of E9.5 wild type or EC-N1ICD embryos (from a cross with *Flk1-Cre *mice) and RNA was isolated from the purified cells for gene expression analysis via real time PCR. To confirm the enrichment of sorted endothelial cells within the PECAM1+ population, expression of lineage markers were compared between RNA from purified PECAM1+ cells and from whole unsorted wild type yolk sac tissue. Endothelial specific genes such as *Cdh5 *(*VE-cadherin*) and *Pecam1 *exhibited significant enrichment in PECAM1+ cells (5.7-fold and 6.7-fold respectively). In contrast, the levels of the primitive visceral endodermal marker *Rhox5 (Pem) *[33] were reduced to 10% in the sorted cells compared to whole wild type yolk sac tissue (Figure 5A), demonstrating a substantial reduction of visceral endoderm cells in the purified endothelial cells. These findings demonstrate a specific enrichment of yolk sac endothelial cells via flow sorting.

The Notch-responsive genes identified in the previous analysis of whole yolk sac tissues were then examined in the sorted yolk sac endothelial cells. The endothelial marker, *Pecam1 *exhibited statistically equivalent expression between the EC-N1CD and wild type sorted cells, similar to that seen in whole yolk sac tissues (data not shown). However, expression of *Hey1 *and *Heyl *was increased 4.4-fold and 16.7-fold in the EC-N1ICD sorted endothelial cells respectively. Similarly, the VEGF family members *Vegfc *and *Pgf *were upregulated 2.1-fold and 1.9-fold, respectively in sorted EC-N1ICD endothelial cells compared to wild type, while *Tgfb2 *was increased 6.9-fold (Figure 5B). These results demonstrate that the expression of these genes is upregulated specifically in the endothelial lineages in EC-N1ICD yolk sac. Taken together, the gene expression defects demonstrate that Notch-Rbpj signaling acts to regulate many key genes specifically in the endothelia of the developing yolk sac. The misexpression of some of these factors may contribute to the vascular differentiation defects seen in the transgenic models.

### Putative Notch regulated genes contain potential RBPJ binding sites in the upstream regulatory region

The in vivo studies have identified a number of putative Notch regulated genes in the developing vasculature. Many of these genes are known Notch targets, including *Hey1 *and *Heyl*, with known RBPJ binding sites within regulatory elements [28]. These studies however also identified altered expression of several genes, including *Vegfc*, *Tgfb2*, and *Pgf*, for which their regulation by Notch signaling has not previously been described. Bioinformatic tools were used to determine if these genes are potentially direct targets of Notch signaling through RBPJ. Several RBPJ binding sites, with a core consensus binding sequence GTGGGAA [34], have been previously identified in known Notch targets such as the HES family members [28,35]. The bioinformatic tool, ECR browser [36], was used to determine if canonical RBPJ binding sites were observed in the promoter proximal regions of loci encoding the secreted factors, *Vegfc*, *Pgf*, and *Tgfb2*. The mouse, human, and rat genomic sequences of each of the target genes were aligned and examined for the RBPJ conserved transcription factor binding sites (TFBS) within the evolutionary conserved regions (ECRs) upstream and downstream of the start site. In each of the genes a GTGGGAA consensus sequence was found, indicating a potential RBPJ site (Additional File 6). In the *Vegfc *locus, an RBPJ binding site is apparent approximately 7.8kb upstream of the start site, while the *Pgf *locus harbors an RBPJ binding site 4.1kb upstream and one 3.8kb downstream of the transcriptional start site. RBPJ sites were observed at approximately 2.5kb and 5.8kb upstream of the start site of the *Tgfb2 *locus. The putative Notch binding sites in each gene suggests that these genes may be direct targets of Notch signaling.

## Discussion

Numerous studies have indicated critical roles for Notch signaling in the proper formation and maintenance of the early vascular system in the mouse. However, the mechanisms by which Notch signaling regulates such diverse aspects of endothelial differentiation in its various contexts are active areas of research. Our analysis details the morphological and molecular defects associated with altered Notch signaling in vivo, with a focus on the extraembryonic vasculature of the yolk sac. This work has characterized distinct morphological defects in the early embryonic and extraembryonic vasculature associated with loss or gain of Notch activity. Substantial gene expression differences in these models point to putative Notch target genes, and suggest potential mechanisms by which Notch signaling directs endothelial cell differentiation.

**Figure 6:**
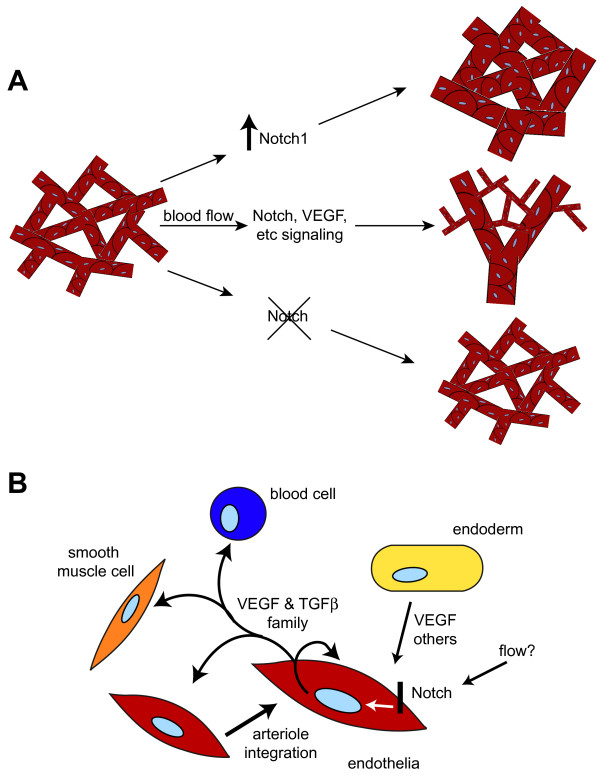
**Notch regulates the expression of key genes and the remodeling of the yolk sac vasculature**. (A) Proposed model depicts the Notch pathway as a key component in the regulation of the remodeling of the vasculature. In wild type yolk sac Notch acts in concert with VEGF and other signaling families to direct the integration of the endothelial cells into both large and small caliber vessels. When Notch is activated this integration is increased leading to very large caliber vessels with limited intra-vascular space. When Notch activity is abrogated the integration is limited and the vascular retains the simple unremodeled appearance of the early vascular plexus. (B) Proposed model of signaling in the endodermal and endothelial cells of the early yolk sac. Signaling from the endoderm and regulation from blood flow activates Notch signaling in select endothelial cells. Notch activates the expression of select secreted genes that act in both a paracrine and juxtacrine manner to direct endothelial cell migration and integration within the developing vasculature.

Morphological analysis of both EC-N1ICD and EC-Rbpj-KO models confirmed that the regulation of Notch signaling is critical in the formation of the intra- and extraembryonic vasculature of the developing embryo. Detailed examination of the yolk sac vasculature, dorsal aortae, and fetal vasculature of the placenta revealed an enlarged vessel phenotype in the EC-N1ICD, remarkably apparent within the yolk sac, in which the plexus is converted to a mass of large diameter vessels (Figure 6A). In contrast, small caliber non-remodeled vessels are present in the EC-Rbpj-KO yolk sac model (Figure 6A). Other Notch models have demonstrated malformations in vessel diameter resulting from altered Notch activity, including other models of activated Notch in vivo [18,27]. *Notch1 *deficient mice [15], mice lacking *Rbpj *in the endothelia, and *Dll4^+/- ^*embryos [26] each exhibit collapsed dorsal aortae and a lack of vascular remodeling. In contrast, embryos overexpressing either *Dll4 *[37] or *Notch4 *[17] have enlarged dorsal aortae. Data indicated that the enlargement of the dorsal aortae in mice overexpressing *Dll4 *was due not to a proliferation of endothelial cells, but to the improper migration of these cells [26]. These data point to an important role for Notch signaling to regulate vessel diameter.

Given that Notch signaling is associated with arterial identity [13,14], this function of Notch signaling to control vessel size may represent an aspect of Notch function in the definition of the arteriole. The mechanisms of how Notch and other signaling pathways control vessel size in the various regions of the developing embryo are not well defined, and are likely context dependent. Some models suggest that directional cell division is important to dictate whether endothelial cell proliferation within a patent vessel results in an increase in luminal diameter [38]. Other models suggest the recruitment of endothelial or angioblast cells into existing vessels as an important mechanism to increase vessel diameter [39]. The remodeling process of the yolk sac makes extensive use of reallocation of cells during remodeling (discussed below), suggesting complex roles for Notch signaling in controlling endothelial cell behavior during remodeling.

The yolk sac vasculature represents a genetically tractable model to study endothelial differentiation, and the work presented here has made extensive analysis of this tissue. Endothelial differentiation within the yolk sac is initiated as groups of cells from the proximally situated extraembryonic mesoderm condense into blood islands by approximately embryonic day 7.0, which subsequently migrate toward the distal region of the yolk sac. The peripheral cells will differentiate to the endothelial cells lining the vasculature, whereas the inner cells become blood cells. The endothelial cells expand and fuse to form the vascular plexus, which is contiguous with the embryonic vasculature at the onset of blood flow [40]. The capillaries are then remodeled into the hierarchical vascular network of vitelline artery, capillaries, and vitelline vein. Observations of vessel remodeling in the yolk sac, particularly in the chick embryo, suggest that the formation of large diameter vessels is from pre-existing capillary-derived endothelial cells. This process involves the collapse of some capillary microvessels, and the endothelial cells from the capillary are then recruited into the nascent vessels to result in a larger diameter vessel (Figure 2G, asterisk). This mode of remodeling resembles the process of intussusceptive arborization, which has been suggested to be an efficient and rapid mode of angiogenesis in a variety of sites [3]. Although Notch signaling does play a significant role in sprouting angiogenesis [41] in many other contexts, the remodeling of the yolk sac in the early embryo may not utilize a sprouting angiogenesis method. Instead, this early vascular remodeling is a rather distinct vascular mechanism that is not entirely clear and remains to be studied in detail.

Based on this model of endothelial remodeling within the yolk sac plexus, the defects in vessel diameter and the remodeling failure in the Notch models used in this study indicate a possible defect in the reallocation of endothelial cells from capillaries into arterioles, suggesting that Notch plays a significant role in this migration. The phenotype observed in the EC-N1ICD yolk sac vasculature is possibly due to increased mobilization and disorganized recruitment of capillary-derived endothelial cells, resulting in a field of enlarged vessels. Conversely the failure of the yolk sac remodeling in the EC-Rbpj-KO model is due to the abrogation of endothelial cell migration to form the larger caliber vessels. Live imaging of the behavior of endothelial yolk sac cells in the Notch models used in this study will be required to elaborate this model.

A detailed understanding of the downstream effectors of Notch signaling during vascular differentiation in vivo, particularly within the extraembryonic tissues, has been lacking. The molecular analysis of the Notch models presented here identified a variety of gene expression defects associated with altered Notch activity. Both the gain- and loss-of-function in vivo models suggest misregulation of a number of genes. Data indicated that two of the Notch family ligands, *Dll4 *and *Jag1 *were upregulated in EC-N1ICD yolk sac, indicating a possible positive feedback loop for Notch signaling to direct expression of its ligands. There is some precedence for a positive regulatory loop in which Notch regulates the expression of ligands, including *Jag1 *in NIH 3T3 cells [31] and *Dll1 *in glioma cells [32].

Importantly, the gene expression analysis also identified a number of secreted factors whose expression within the endothelia is altered in the Notch models. These data suggest that the Notch signaling pathway regulates a number of secreted factors important in endothelial differentiation such as, *Pgf*, *Vegfc*, and *Tgfb2*, either directly or indirectly. Targeted deletions of select TGF-β signaling components in mice result in the improper formation of the yolk sac vasculature, indicating the importance for TGF-β signaling in the formation of the early vascular system [42]. Vegf signaling has critical early roles in the formation of the blood vessels in the early embryo [43,44]. VEGF has also been shown to act as an upstream component of Notch in the signaling cascade directing the differentiation of the zebrafish vasculature [45]. PlGF has known roles in pathological angiogenesis [46,47], and may act as a VEGF agonist by countering the effects of the VEGFA antagonist Flt1. In addition to the essential functions of VEGFC in the developmental origin of the lymphatic system [48,49], there is conflicting data to suggest it plays a broader role in angiogenesis. Although this factor has known angiogenic activities in certain assays [50], no vascular defects have been reported in mice lacking *Vegfc *[48]. The expression of VEGFC within the yolk sac however suggests a potential non-lymphatic function for this factor in this tissue. A putative role for VEGFC in angiogenesis may involve the modulation of its receptors, Kdr (VEGFR2) and Flt4 (VEGFR3), both of which play a critical role in early angiogenesis.

Notch signaling is essential for vessel remodeling, as our data shows that Notch regulates the expression of a variety of secreted factors. Notch signaling may play potential nonautonomous roles in the remodeling of the yolk sac capillary plexus (Figure 6B). Notch signaling is important for the regulation of select signaling molecules, including members of the VEGF family and TGF-β family, which would emanate from the developing arteriole where activity of Notch signaling is highest. These molecules then act in a paracrine and autocrine manner to elaborate the local arterial microenvironment about Notch expressing cells, which may potentially influence adjacent capillary endothelial cells, smooth muscle or mural cells, and hematopoietic cells. Indeed, nonautonomous functions for Notch signaling in the endothelium have been suggested, including attenuating proliferation of smooth muscle cells [27]. Testing of the nonautonomous functions of Notch signaling in the vasculature will require determining the roles of Notch-regulated secreted factors in vivo. These experiments may include the use of conditional transgenics and knockouts of these factors in the endothelia, and determining remodeling and endothelial differentiation defects in these models.

Yolk sac vessel remodeling occurs after the initiation of blood flow, which initiates at approximately E8.5 in the mouse [40], and this flow is essential for the remodeling process. It is formally possible that the enlarged luminal diameter of the dorsal aorta in the Notch gain-of-function model could give rise to a secondary failure of yolk sac remodeling solely due to reduced flow and associated shear stress. However, the vessel architecture reported in mouse models with remodelling defects solely due to altered blood flow [40] display very different phenotypes from those observed in the N1ICD model. This comparison suggests that the remodeling defects and some of the associated gene expression defects in the yolk sac in this Notch1 gain-of-function model are not secondary to embryonic vessel defects or defects in blood flow. The cellular and subsequent molecular response to the shear stress associated with blood flow is not completely understood, but may involve a complex cell surface signalling molecules including KDR, PECAM1, and VE-cadherin [51]. Shear stress thus initiates a molecular cascade in endothelial cells to direct further morphological changes to direct remodeling. An important question is what is the link between blood flow and Notch signaling, both of which are essential for vessel remodeling. During remodeling, an arteriole rudiment is first observed at the site of yolk sac vasculature contact with the omphalomesentric artery. Early *Dll4 *expression is observed at this arteriole rudiment within the yolk sac [52], indicating it is likely the Notch ligand that initiates Notch activity within this restricted region of the yolk sac vasculature. It remains to be seen the extent to which fluid dynamics control the expression of Notch signalling components and the subsequent remodeling process.

## Conclusions

The transcriptional network controlled by Notch signaling during extraembryonic endothelial differentiation is largely unknown. The present data demonstrate a role for Notch signaling in the regulation of a number of key genes in the embryonic vasculature of the yolk sac, including a variety of secreted factors important for endothelial differentiation. These downstream targets suggest a mechanism for Notch regulation of vessel diameter size during the remodeling process in the yolk sac vasculature. Further work on the in vivo models will help to further define the relationship between the transcriptional networks regulated by Notch to direct endothelial differentiation, and the role of these Notch downstream targets in endothelial migration and vascular remodeling. The understanding of these interactions and processes will aid in the development of treatments affecting vascular differentiation, including heart disease and tumor progression.

## Methods

### Mice and embryos

The generation of *Rosa^Notch ^*mice, *loxP*-flanked *Rbpj*, *Tie2-Cre *and *Flk1-Cre *mice have been described previously [21-24]. *Tie2-Cre *mice and *loxP*-flanked *Rbpj *mice were mated to obtain *Tie2-Cre; Rbpj^f/+ ^*males. Breeding pairs of *Tie2-Cre *or *Flk1-Cre *mice and *Rosa^Notch ^*mice or *Tie2-Cre; Rbpj^f/+ ^*males and *Rbpj^f/+ ^*females were intercrossed and the presence of a vaginal plug was identified as E0.5. Embryos were dissected from the decidual tissue at E8.5, E9.5, or E10.5 and treated according to the intended protocol. Placenta were separated from the embryo along with the mesometrial portion of the decidua and prepared for histology. All experimental procedures were performed with prior approval of the University Institutional Animal Care and Use Committee in accordance with guidelines established by the American Veterinary Medical Association.

### Genotyping of Progeny

The genotypes of all offspring were analyzed by PCR on genomic DNA isolated from ear punches, yolk sac samples, or embryonic tissue depending on the intended protocol. Genomic DNA was isolated using the DNeasy Blood & Tissue Kit (Qiagen). The *Tie2-Cre *and *Flk1-Cre *transgene presence was tested with the primers Cre-R3, 5'-AAT GCT TCT GTC CGT TTG-3' and Cre-F3, 5'-GGA TTA ACA TTC TCC CAC C-3', giving a 458-bp band [53]. PCR genotyping for *Rosa^Notch ^*mice was performed as described [25]. The wild type, floxed, and deleted *Rbpj *alleles were genotyped as described [54].

### Immunochemistry

Embryos with or without the surrounding yolk sac were fixed in 4% paraformaldehyde/PBS overnight at 4°C. The following day they were rinsed in PBS, dehydrated in methanol, and bleached in 1mL of 1.5% H_2_O_2 _in methanol for 4-5 hours at room temperature. Embryos were then rehydrated through methanol into PBS, blocked in PBSMT (PBS, 3% nonfat dry milk, 0.1% Triton X-100) for 2 hours at room temperature and incubated with 1:50 anti-PECAM1 antibody (BD Pharmingen) overnight at 4°C. Embryos were washed 5 times for 1 hour each with PBSMT and incubated with 1:100 HRP-coupled anti-rat IgG (#14-16-12, kpl.com) overnight at 4°C. Embryos were again washed 5 times in PBSMT with a final wash in PBT (PBS, 0.2% BSA, 0.1% Triton X-100). Then embryos were incubated in developing solution (0.3 mg/mL DAB, 0.5% NiCl_2 _in PBT) for 20 minutes at room temperature followed by the addition of H_2_O_2 _at a 0.03% final concentration. Once the color developed (approximately 10min.), the embryos were rinsed in PBT and then PBS and fixed overnight in 2% paraformaldehyde/0.1% gluteraldehyde/PBS at 4°C. Embryos were then rinsed in PBS and either equilibrated into 70% glycerol for imaging or dehydrated in ethanol for paraffin embedding.

### X-gal staining

Untreated whole-mount E8.0 and E8.5 embryos with surrounding yolk sac were fixed on ice for 10 min in a fixation solution. The samples were then washed 3 times in 1X PBS pH 7.4 and stained overnight at 37°C per established procedures [55]. The next day, the samples were rinsed in PBS and fixed overnight in a 3.7% paraformaldehyde/PBS solution. The following day, the samples were again rinsed, imaged and stored in 70% EtOH for paraffin embedding.

### Histology

Untreated whole-mount embryos, whole-mount PECAM1 stained embryos and placenta were fixed in 3% paraformaldehyde overnight, dehydrated into 70% ethanol and embedded in paraffin wax. Embryos were then sectioned on the transverse generally in 8 μm sections. For histological observation, Hematoxylin and Eosin or Nuclear Fast Red staining was conducted on the paraffin sections and sections were observed.

### Preparation of single yolk sac cell suspension

E9.5 embryos were obtained from timed matings between *Flk1-Cre *females and *Rosa^Notch ^*males. Yolk sacs were incubated in 0.1% collagenase (StemCell Technologies Inc)/phosphate-buffered saline (PBS)/20% fetal bovine serum at 37°C for 30 minutes. The digested yolk sacs were aspirated through 27-gauge needles to fully separate the cells. Genotyping was performed on DNA isolated from corresponding embryo tissues.

### Cell sorting with PECAM1 and analysis of PECAM1+ cells

Single-cell suspensions from yolk sacs were incubated for 30 minutes at 4°C with anti-PECAM1 antibody conjugated to phycoerythrin cyanine 7 (eBioscience Inc). The PECAM1+ cells were isolated by cell sorting with the use of a BD FACSAria cell sorter (BD Biosciences). Total RNA was isolated from the sorted cells using an RNeasy mini kit (Qiagen) and RNA was used for real time PCR analysis.

### RT-PCR analysis

Embryos were dissected at E9.5. The uterus and decidua were carefully removed and discarded. The yolk sac was separated from the embryo and the embryo was used for genotyping. Total RNA was isolated from the yolk sac using an RNeasy mini kit (Qiagen). cDNA was generated using SuperScript III reverse transcriptase (Invitrogen). Semiquantitative RT-PCR was performed using a number of primers from IDT (idtdna.com) (Additional File 7), with ribosomal protein L7 (5'-GAA GCT CAT CTA TGA GAA GGC-3' and 5'-AAG ACG AAG GAG CTG CAG AAC-3') as a control. The annealing temperature and number of PCR cycles was optimized for each reaction.

### Real Time PCR

RNA was isolated from sorted endothelial cells using an RNeasy mini kit (Qiagen). cDNA from endothelial cells was generated using Superscrpit III reverse transcriptase (Invitrogen) and quantitative real-time PCR analysis was performed using Taqman primer sets with the 7500 Real Time PCR system (Applied Biosystems). Gene expression was normalized to GAPDH. Specific ABI Taqman primer/probe assay IDs are available upon request.

### Microarray Analysis

RNA was isolated from yolk sac tissues using an RNeasy mini kit (Qiagen). RNA was initially analyzed with the Mouse Genome 430 A Array from Affymetrix. Microarray data was deposited to the Gene Expression Omnibus (GSE22418).

### Yolk sac immunofluorescence

Embryos were dissected at E9.5 in PBS. The uterus and decidua were carefully removed and discarded. The yolk sac was separated from the embryo by severing the vitelline arteries and the embryo was used for genotyping. The yolk sacs were collected in separate wells of a 24 well plate and then fixed in 1mL of 3% paraformaldehyde/PBS for 15 minutes on ice. Then the yolk sacs were permeabilized in 1mL of PBS containing 0.02% Triton X-100 for 30 minutes. Yolk sacs were transferred to separate 1.5mL Epindorf tubes containing 500uL of blocking solution (PBS, 3% BSA, 5% donkey serum (Jackson ImmunoResearch)) for 2 hours at room temperature. Blocking solution was changed once at 1 hour. Yolk sacs were then incubated with 1:33 anti-PECAM1 antibody (#557355, BD Pharmingen) overnight at 4°C. Embryos were washed 5 times for 1 hour each with blocking solution and incubated with 1:75 FITC-conjugated AffiniPure Anti-Rat IgG (#712-095-153, Jackson ImmunoResearch) overnight at 4°C. Embryos were again washed 5 times in blocking solution with a final 20-minute wash in PBS. The yolk sacs were transferred via Pasteur pipette to a drop of *SlowFade *Gold antifade reagent (Molecular Probes) on a glass slide and covered with a cover slip.

### Histology Immunofluorescence

Embryos were prepared following the above protocol for histology. Tissues were deparrafinized through xylene and EtOH into cold tap water. Antigen retrieval was performed by immersing the slides in 10 mM calcium citrate for 20 minutes in a steam chamber. Sections were washed first with water and then 2 times with PBS. Sections were then incubated in PBT (PBS, 0.2% BSA, 0.1% Triton X-100) for 2 hours at room temperature. Sections were stained with a-smooth muscle actin (1:250 dilution; catalog no. A2547; Sigma-Aldrich, Inc) for 1 hour at 4°C. Sections were washed 3 times with PBT and then stained with Alexa Fluor 546 goat anti-mouse IgG (1:250 dilution; catalog no. A11003; Invitrogen) for 30 minutes at room temperature in the dark. Sections were again washed 3 times with blocking solution. A small amount of ProLong Gold antifade reagent with DAPI (Invitrogen) was applied and the samples were covered with a cover slip, allowed to set, and imaged. Slides were stored at -20°C.

### Identification of TFBSs

The ECR browser [36] was used to determine the location of potential RBPJ binding sites. The evolutionary conserved regions of the mouse, human, and rat were examined upstream and flanking the transcription start site of each gene for the presence of the RBPJ binding sequence (GTGGGAA) [34].

### Microscopy and image acquisition

Images were acquired with a Nikon SMZ800 dissecting microscope for whole embryos and a Nikon ECLIPSE 55i for embryonic sections using a Leica DFC480 camera and Leica FireCam 3.0 software. Images for yolk sac immunofluorescence were acquired with an Olympus 1X71 microscope and Olympus DP71 camera using Olympus DP71 controller software. Adobe Photoshop CS2 was used for photograph editing.

### Statistical Analysis

Data bars represent the means +/- standard error of the mean. RNA analyses of yolk sac tissues were performed with an n of 5. The statistical significance of the data was determined using a t-test with a p-value of < 0.05 considered statistically significant.

## Authors' contributions

JLV conceived the project, obtained funding, participated in the design of the experiments, and co-wrote the manuscript; JNC participated in the design of the experiments, performed experiments, analyzed data and co-wrote the manuscript. YF and PEF designed and assisted in the yolk sac cell suspension experiment. NKN carried out the *Flk1-Cre *lacZ experiments. All authors read and approved the final manuscript.

## Supplementary Material

Additional file 1 ***Tie2-Cre *and *Flk1-Cre *are expressed in the endothelia of the early embryo**. (A-E) X-Gal staining of embryos from R26R cross with *Tie2-Cre *transgene. (A-C) Whole mount E8.5 embryos. (D, E) Histological sections of E8.5 embryos. (F-I) X-Gal staining of embryos from R26R cross with *Flk1-Cre *transgene. (F) Whole mount E8.0 embryo with surrounding yolk sac. (G) Whole mount E8.5 embryo. (H) Yolk sac from a E8.5 embryo. (I) Histological section of E8.5 embryo. Note expression in the endothelia of dorsal aorta (DA), heart (He), anterior cardinal vein (ACV), yolk sac (YS), allantois (Al), lateral plate (LP), and vitelline artery (VA). Click here for file

Additional file 2 **Embryonic growth and vascular remodeling is normal in early EC-N1ICD embryos**. (A, B) Whole mount E8.5 wild type (A) and EC-N1ICD littermate (B) embryos with surrounding yolk sac stained with an antibody to PECAM1. EC-N1ICD yolk sac vasculature appeared normal. (C, D) Lateral view of E8.5 wild type embryo (C) and EC-N1ICD littermate (D) stained with an antibody to PECAM1. EC-N1ICD embryos appeared normal. Scale bars are 500 μm (A, B) and 250 μm (C, D). Click here for file

Additional file 3 **Gene expression in EC-N1ICD and EC-Rbpj-KO yolk sac tissues**. A graphical representation of possible outcomes of expression data and the corresponding genes that display this type of expression. Click here for file

Additional file 4 Expression of genes encoding secreted factors in EC-N1ICD and EC-Rbpj-KO yolk sac tissues Click here for file

Additional file 5 **Histograms obtained from PECAM1-PE Cy7 fluorescent activated cell sorting**. Representative histograms showing the distribution of dissociated yolk sac cells for the (A) isotype control and PECAM1 stained (B) wild type yolk sac and (C) EC-N1ICD yolk sac. The gating used to purify PECAM1+ cells is indicated. Click here for file

Additional file 6 **rVista visualization of conserved RBPJ binding sites**. Using the ECR browser, the genomic sequence of each of the three secreted genes, *Vegfc*, *Pgf*, and *Tgfb2 *was examined for the RBPJ binding site. The red bars identify the resulting binding sites. Click here for file

Additional file 7 Primer pairs used for RT-PCR Click here for file

## References

[B1] Risau W Flamme I Vasculogenesis Annu Rev Cell Dev Biol 1995 11 73 91 10.1146/annurev.cb.11.110195.000445 8689573

[B2] Drake CJ Fleming PA Vasculogenesis in the day 6.5 to 9.6 mouse embryo Blood 2000 95 1671 1679 10688823

[B3] Djonov V Schmid M Tschanz SA Burri PH Intussusceptive Angiogenesis: its role in embryonic vascular network formation Circ Res 2000 86 286 10679480 10.1161/01.res.86.3.286

[B4] Risau W Mechanisms of angiogenesis Nature 1997 386 671 674 10.1038/386671a0 9109485

[B5] Patan S Vasculogenesis and angiogenesis as mechanisms of vascular network formation, growth and remodeling J Neurooncol 2000 50 1 15 10.1023/A:1006493130855 11245270

[B6] Jain RK Molecular regulation of vessel maturation Nat Med 2003 9 685 693 10.1038/nm0603-685 12778167

[B7] Carmeliet P Mechanisms of angiogenesis and arteriogenesis Nat Med 2000 6 389 395 10.1038/74651 10742145

[B8] Tallquist M Soriano P Klinghoffer RA Growth factor signaling pathways in vascular development Oncogene 1999 18 7917 7932 10.1038/sj.onc.1203216 10630644

[B9] Yancopoulos GD Davis S Gale NW Rudge JS Wiegand SJ Holash J Vascular-specific growth factors and blood vessel formation Nature 2000 407 242 248 10.1038/35025215 11001067

[B10] Krebs LT Xue Y Norton CR Shutter JR Maguire M Sundberg JP Gallahan D Closson V Kitajewski J Callahan R Smith GH Stark KL Gridley T Notch signaling is essential for vascular morphogenesis in mice Genes Dev 2000 14 1343 1352 10837027 PMC316662

[B11] Artavanis-Tsakonas S Rand MD Lake RJ Notch signaling: cell fate control and signal integration in development Science 1999 284 770 776 10.1126/science.284.5415.770 10221902

[B12] Mumm JS Kopan R Notch signaling: from the outside in Dev Biol 2000 228 151 165 10.1006/dbio.2000.9960 11112321

[B13] Uyttendaele H Marazzi G Wu G Yan Q Sassoon D Kitajewski J Notch4/int-3, a mammary proto-oncogene, is an endothelial cell-specific mammalian Notch gene Development 1996 122 2251 2259 8681805 10.1242/dev.122.7.2251

[B14] Villa N Walker L Lindsell CE Gasso J Iruela-Arispe ML Weinmaster G Vascular expression of Notch pathway receptors and ligands is restricted to arterial vessels Mech Dev 2001 108 161 164 10.1016/S0925-4773(01)00469-5 11578869

[B15] Swiatek PJ Lindsell CE del Amo FF Weinmaster G Gridley T Notch1 is essential for postimplantation development in mice Genes Dev 1994 8 707 719 10.1101/gad.8.6.707 7926761

[B16] Iso T Hamamori Y Kedes L Notch signaling in vascular development Arterioscler Thromb Vasc Biol 2003 23 543 553 10.1161/01.ATV.0000060892.81529.8F 12615665

[B17] Uyttendaele H Ho J Rossant J Kitajewski J Vascular patterning defects associated with expression of activated Notch4 in embryonic endothelium Proc Natl Acad Sci USA 2001 98 5643 5648 10.1073/pnas.091584598 11344305 PMC33266

[B18] Krebs LT Starling C Chervonsky AV Gridley T *Notch1 *activation in mice causes arteriovenous malformations phenocopied by ephrinB2 and EphB4 mutants Genesis 2010 48 146 150 20101599 10.1002/dvg.20599 PMC2849749

[B19] Shawber CJ Funahashi Y Francisco E Vorontchikhina M Kitamura Y Stowell SA Borisenko V Feirt N Podgrabinska S Shiraishi K Chawengsaksophak K Rossant J Accili D Skobe M Kitajewski J Notch alters VEGF responsiveness in human and murine endothelial cells by direct regulation of VEGFR-3 expression J Clin Invest 2007 117 3369 3382 10.1172/JCI24311 17948123 PMC2030453

[B20] Funahashi Y Shawber CJ Vorontchikhina M Sharma A Outtz HH Kitajewski J Notch regulates the angiogenic response via induction of VEGFR-1 J Angiogenes Res 2010 2 3 10.1186/2040-2384-2-3 20298529 PMC2828996

[B21] Murtaugh LC Stanger BZ Kwan KM Melton DA Notch signaling controls multiple steps of pancreatic differentiation Proc Natl Acad Sci USA 2003 100 14920 14925 10.1073/pnas.2436557100 14657333 PMC299853

[B22] Tanigaki K Han H Yamamoto N Tashiro K Ikegawa M Kuroda K Suzuki A Nakano T Honjo T Notch-RBP-J signaling is involved in cell fate determination of marginal zone B cells Nat Immunol 2002 3 443 450 10.1038/ni793 11967543

[B23] Koni PA Joshi SK Temann UA Olson D Burkly L Flavell RA Conditional vascular cell adhesion molecule 1 deletion in mice: impaired lymphocyte migration to bone marrow J Exp Med 2001 193 741 754 10.1084/jem.193.6.741 11257140 PMC2193418

[B24] Motoike T Markham DW Rossant J Sato TN Evidence for novel fate of Flk1^+ ^progenitor: Contribution to muscle lineage Genesis 2003 35 153 159 10.1002/gene.10175 12640619

[B25] Soriano P Generalized lacZ expression with the ROSA26 Cre reporter strain Nat Genet 1999 21 70 71 10.1038/5007 9916792

[B26] Krebs LT Shutter JR Tanigaki K Honjo T Stark KL Gridley T Haploinsufficient lethality and formation of arteriovenous malformations in Notch pathway mutants Genes Dev 2004 18 2469 2473 10.1101/gad.1239204 15466160 PMC529533

[B27] Venkatesh DA Park K Harrington A Miceli-Libby L Yoon JK Liaw L Cardiovascular and hematopoietic defects associated with Notch1 activation in embryonic Tie2-expressing populations Circ Res 2008 103 423 431 10.1161/CIRCRESAHA.108.177808 18617694 PMC2654335

[B28] Maier MM Gessler M Comparative analysis of the human and mouse Hey1 promoter: Hey genes are new Notch target genes Biochem Biophys Res Commun 2000 275 652 660 10.1006/bbrc.2000.3354 10964718

[B29] Radtke F Raj K The role of Notch in tumorigenesis: oncogene or tumour suppressor? Nat Rev Cancer 2003 3 756 767 10.1038/nrc1186 14570040

[B30] Bolos V Grego-Bessa J de la Pompa JL Notch signaling in development and cancer Endocrine Rev 2007 28 339 363 10.1210/er.2006-0046 17409286

[B31] Ross DA Kadesh T Consequences of Notch-mediated induction of Jagged1 Exp Cell Res 2004 296 173 182 10.1016/j.yexcr.2004.02.003 15149848

[B32] Qian C Yan W Zhang J Shi L Qian J Fu Z Kang C Liu N You Y Notch1 induces enhanced expression of D-like-1 in the U251MG glioma cell line Int J Mol Med 2009 24 445 451 19724883 10.3892/ijmm_00000251

[B33] Lin TP Labosky PA Grabel LB Kozak CA Pitman JL Kleeman J MacLeod CL The Pem homeobox gene is X-linked and exclusively expressed in extraembryonic tissues during early murine development Dev Biol 1994 166 170 179 10.1006/dbio.1994.1305 7958444

[B34] Tun T Hamaguchi Y Matsunami N Furukawa T Honjo T Kawaichi M Recognition sequence of a highly conserved DNA binding protein RBP-J kappa Nucleic Acids Res 1994 22 965 971 10.1093/nar/22.6.965 8152928 PMC307916

[B35] Nishimura M Fumiaki I Makoto I Tomita K Tsuda H Nakanishi S Kageyama R Structure, chromosomal locus, and promoter of mouse Hes2 gene, a homologue of Drosophila hairy and Enhancer of split Genomics 1998 49 69 75 10.1006/geno.1998.5213 9570950

[B36] Ovcharenko I Nobrega MA Loots GG Stubbs L ECR browser: a tool for visualizing and accessing data from comparisons of multiple vertebrate genomes Nucleic Acids Res 2004 32 W280 286 10.1093/nar/gkh355 15215395 PMC441493

[B37] Trindade A Kumar SR Scehnet JS Lopes-da-Costa L Becker J Jiang W Liu R Gill PS Duarte A Overexpression of delta-like 4 induces arterialization and attenuates vessel formation in developing mouse embryos Blood 2008 112 1720 1729 10.1182/blood-2007-09-112748 18559979 PMC2518882

[B38] Zeng G Taylor SM McColm JR Kappas NC Kearney JB Williams LH Hartnett ME Bautch VL Orientation of endothelial cell division is regulated by VEGF signaling during blood vessel formation Blood 2007 109 1345 1352 10.1182/blood-2006-07-037952 17068148 PMC1794069

[B39] Schmidt A Brixius K Bloch W Endothelial precursor cell migration during vasculogenesis Circ Res 2007 101 125 136 10.1161/CIRCRESAHA.107.148932 17641236

[B40] Lucitti JL Jones EAV Huang C Chen J Fraser SE Dickinson ME Vascular remodeling of the mouse yolk sac requires hemodynamic force Dev 2007 134 3317 3326 10.1242/dev.02883 PMC4260474 17720695

[B41] Siekmann AF Lawson ND Notch signaling and the regulation of angiogenesis Cell Adh Migr 2007 1 104 106 10.4161/cam.1.2.4488 19329884 PMC2633979

[B42] Goumans MJ Mummery C Functional analysis of the TGF receptor/Smad pathway through gene ablation in mice Int J Dev Biol 2000 44 253 265 10853822

[B43] Ferrara N Davis-Smyth T The biology of vascular endothelial growth factor Endocrine Rev 1997 18 4 25 10.1210/er.18.1.4 9034784

[B44] Carmeliet P Ferreira V Breier G Pollefeyt S Kieckens L Gertsenstein M Fahrig M Vandenhoeck A Harpal K Eberhardt Cm Declercq C Pawling J Moons L Collen D Risau W Nagy A Abnormal blood vessel development and lethality in embryos lacking a single VEGF allele Nature 1996 380 435 439 10.1038/380435a0 8602241

[B45] Lawson ND Vogel AM Weinstein BM Sonic hedgehog and vascular endothelial growth factor act upstream of the Notch pathway during arterial endothelial differentiation Dev Cell 2002 3 127 136 10.1016/S1534-5807(02)00198-3 12110173

[B46] Carmeliet P Moons L Luttun A Vincenti V Compernolle V De Mol M Wu Y Bono F Devy L Beck H Scholz D Acker T DiPalma T Dewerchin M Noel A Stalmans I Barra A Blacher S Vandendriessche T Ponten A Eriksson U Plate KH Foidart JM Schaper W Charnock-Jones DS Hicklin DJ Herbert JM Collen D Persico MG Synergism between vascular endothelial growth factor and placental growth factor contributes to angiogenesis and plasma extravasation in pathological conditions Nat Med 2001 7 575 583 10.1038/87904 11329059

[B47] Oura H Bertoncini J Velasco P Brown LF Carmeliet P Detmar M A critical role of placental growth factor in the induction of inflammation and edema formation Blood 2003 101 560 567 10.1182/blood-2002-05-1516 12393422

[B48] Karkkainen MJ Haiko P Sainio K Partanen J Taipale J Petrova TV Jeltsch M Jackson DG Talikka M Rauvala H Betsholtz C Alitalo K Vascular endothelial growth factor C is required for sprouting of the first lymphatic vessels from embryonic veins Nat Immunol 2004 5 74 80 10.1038/ni1013 14634646

[B49] Lohela M Bry M Tammela T Alitalo K VEGFs and receptors involved in angiogenesis versus lymphangiogenesis Curr Opin Cell Biol 2009 21 154 165 10.1016/j.ceb.2008.12.012 19230644

[B50] Cao Y Linden P Farnebo J Cao R Eriksson A Kumar V Qi JH Claesson-Welsh L Alitalo K Vascular endothelial growth factor C induces angiogenesis in vivo Proc Natl Acad Sci USA 1998 95 14389 14394 10.1073/pnas.95.24.14389 9826710 PMC24383

[B51] Tzima E Irani-Tehrani M Kiosses WB Dejana E Schultz DA Engelhardt B Cao G DeLisser H Schwartz MA A mechanosensory complex that mediates the endothelial cell response to fluid shear stress Nature 2005 437 426 431 10.1038/nature03952 16163360

[B52] Duarte A Hirashima M Benedito R Trindade A Diniz P Bekman E Costa L Henrique D Rossant J Dosage-sensitive requirement for mouse Dll4 in artery development Genes Dev 2004 18 2474 2478 10.1101/gad.1239004 15466159 PMC529534

[B53] Steenhard BM Isom K Stroganova L St John PL Freeburg PB Holzman LB Abrahamson DR Deletion of Von Hippel-Lindau in glomerular podocytes results in glomerular basement membrane thickening, ectopic subepithelial deposition of collage α1α2α1(IV), expression of neuroglobin, and proteinuria Am J Pathol 2010 177 84 96 10.2353/ajpath.2010.090767 20522651 PMC2893653

[B54] Souilhol C Cormier S Tanigaki K Babinet C Cohen-Tannoudji M RBP-Jkappa-dependent notch signaling is dispensable for mouse early embryonic development Mol Cell Biol 2006 26 4769 4774 10.1128/MCB.00319-06 16782866 PMC1489163

[B55] Venuti JM Morris JH Vivian JL Olson EN Klein WH Myogenin is required for late by not early aspects of myogenesis during mouse development J Cell Biol 1995 128 563 576 10.1083/jcb.128.4.563 7532173 PMC2199898

