# Supplementary figures and images for: Notch signaling regulates remodeling and vessel diameter in the extraembryonic yolk sac
Source: BMC Dev Biol. 2011 Feb 25;11:12. doi: 10.1186/1471-213X-11-12 (PMC3051915; doi:10.1186/1471-213X-11-12)

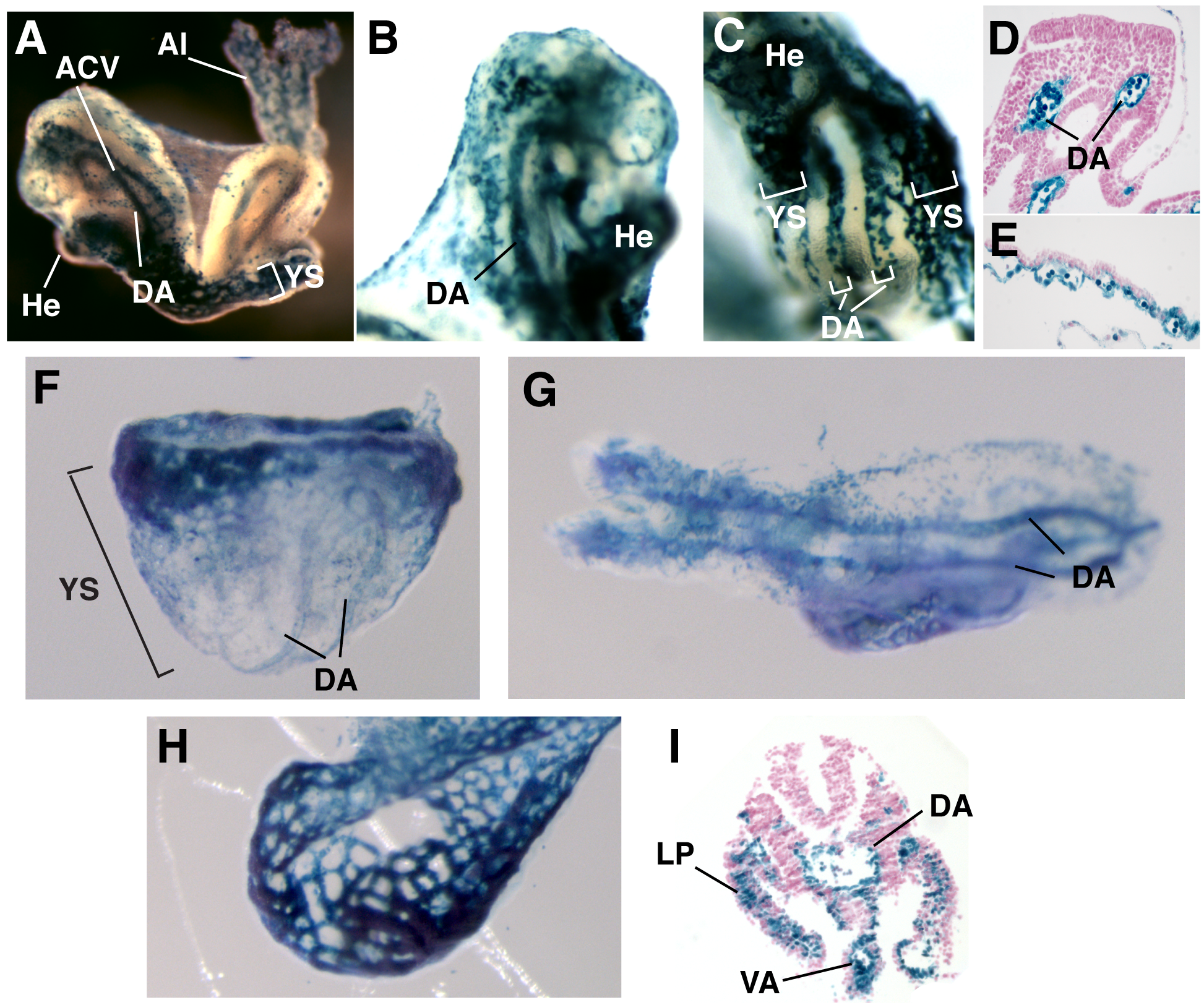

Supplement: Additional file 1 — Tie2-Cre and Flk1-Cre are expressed in the endothelia of the early embryo. (A-E) X-Gal staining of embryos from R26R cross with Tie2-Cre transgene. (A-C) Whole mount E8.5 embryos. (D, E) Histological sections of E8.5 embryos. (F-I) X-Gal staining of embryos from R26R cross with Flk1-Cre transgene. (F) Whole mount E8.0 embryo with surrounding yolk sac. (G) Whole mount E8.5 embryo. (H) Yolk sac from a E8.5 embryo. (I) Histological section of E8.5 embryo. Note expression in the endothelia of dorsal aorta (DA), heart (He), anterior cardinal vein (ACV), yolk sac (YS), allantois (Al), lateral plate (LP), and vitelline artery (VA). [file 1471-213X-11-12-S1.PNG]

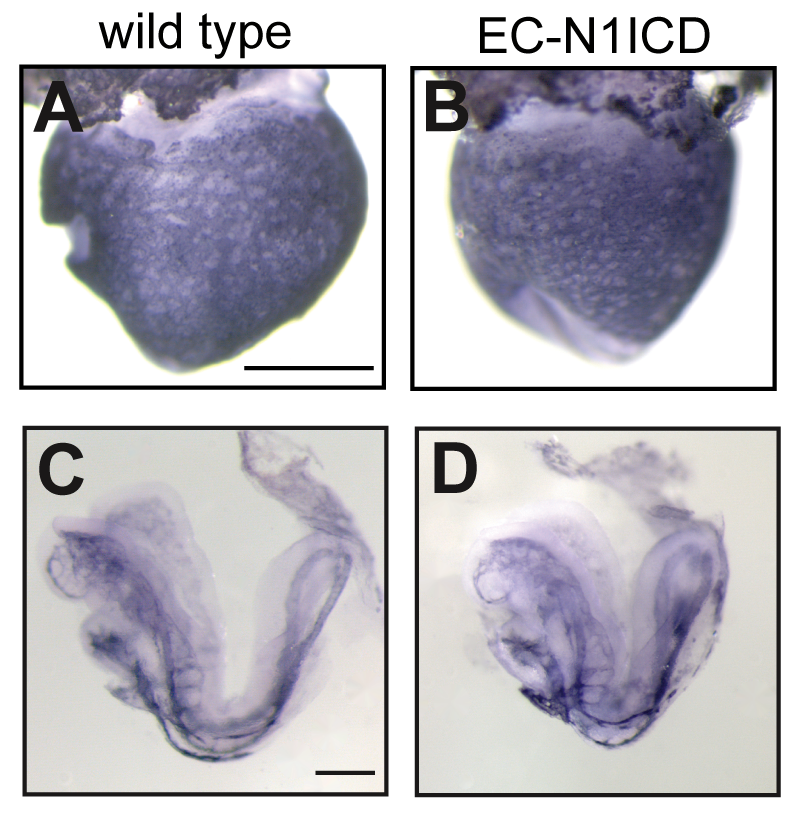

Supplement: Additional file 2 — Embryonic growth and vascular remodeling is normal in early EC-N1ICD embryos. (A, B) Whole mount E8.5 wild type (A) and EC-N1ICD littermate (B) embryos with surrounding yolk sac stained with an antibody to PECAM1. EC-N1ICD yolk sac vasculature appeared normal. (C, D) Lateral view of E8.5 wild type embryo (C) and EC-N1ICD littermate (D) stained with an antibody to PECAM1. EC-N1ICD embryos appeared normal. Scale bars are 500 μm (A, B) and 250 μm (C, D). [file 1471-213X-11-12-S2.PNG]

**A**

isotype control  
Specimen\_001-J14-2

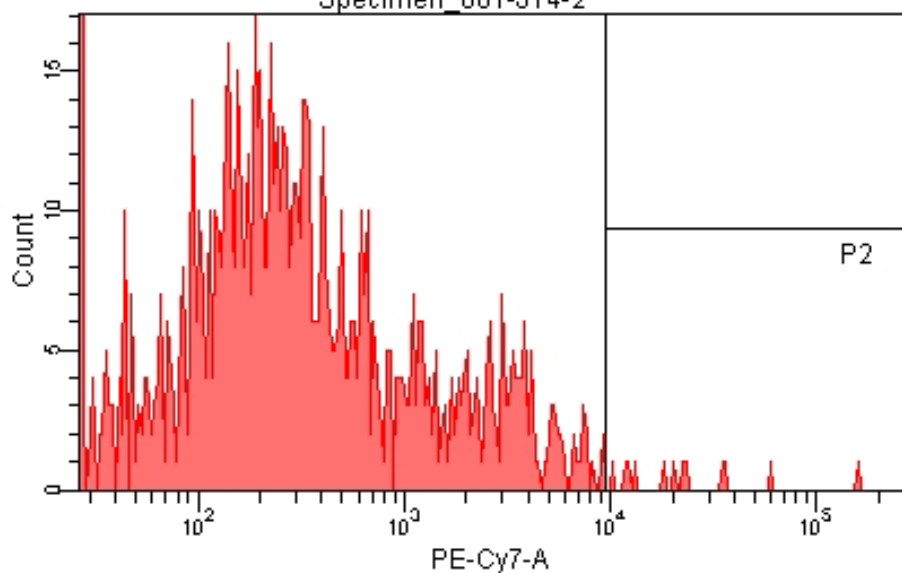**B**

wild type  
Specimen\_001-J14-5

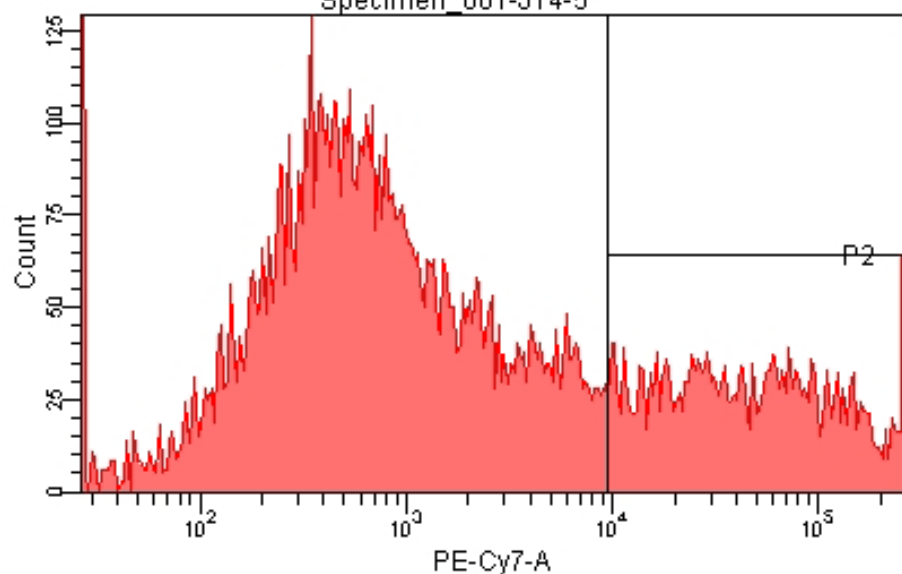**C**

EC-N1ICD  
Specimen\_001-J14-4

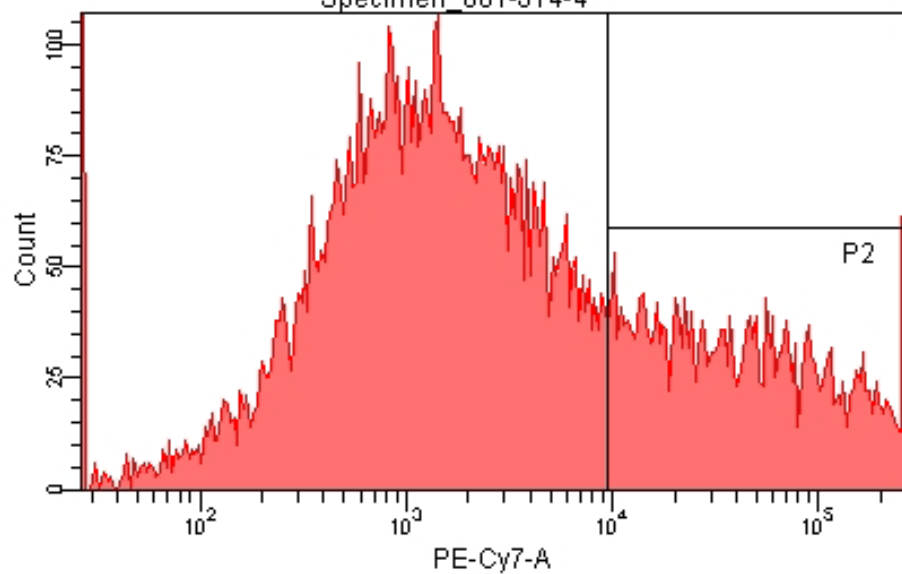

Supplement: Additional file 5 — Histograms obtained from PECAM1-PE Cy7 fluorescent activated cell sorting. Representative histograms showing the distribution of dissociated yolk sac cells for the (A) isotype control and PECAM1 stained (B) wild type yolk sac and (C) EC-N1ICD yolk sac. The gating used to purify PECAM1+ cells is indicated. [file 1471-213X-11-12-S5.PDF]

*Vegfc*

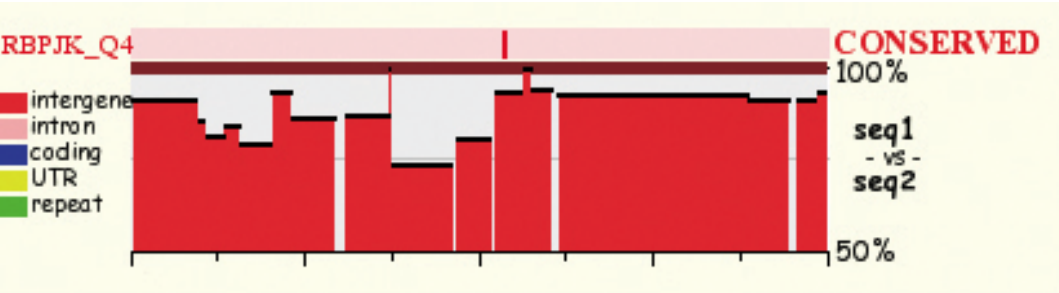

*Pgf*

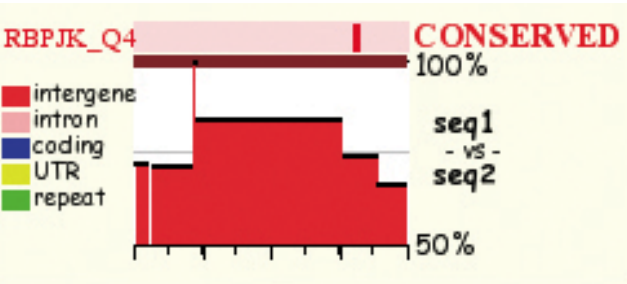

*Tgfb2*

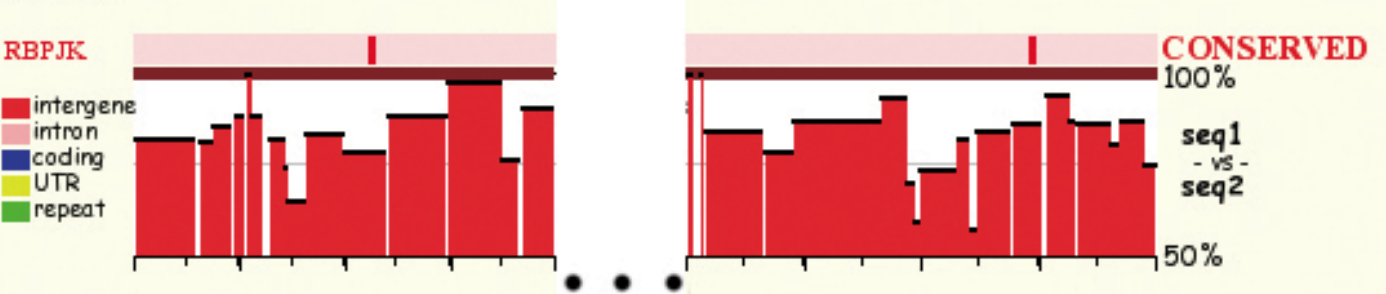

Supplement: Additional file 6 — rVista visualization of conserved RBPJ binding sites. Using the ECR browser, the genomic sequence of each of the three secreted genes, Vegfc, Pgf, and Tgfb2 was examined for the RBPJ binding site. The red bars identify the resulting binding sites. [file 1471-213X-11-12-S6.PDF]
