# Supplementary material for: Notch signaling regulates remodeling and vessel diameter in the extraembryonic yolk sac
Source: BMC Dev Biol. 2011 Feb 25;11:12. doi: 10.1186/1471-213X-11-12 (PMC3051915; doi:10.1186/1471-213X-11-12)
Supplement: Additional file 3 — Gene expression in EC-N1ICD and EC-Rbpj-KO yolk sac tissues. A graphical representation of possible outcomes of expression data and the corresponding genes that display this type of expression. [file 1471-213X-11-12-S3.PDF]

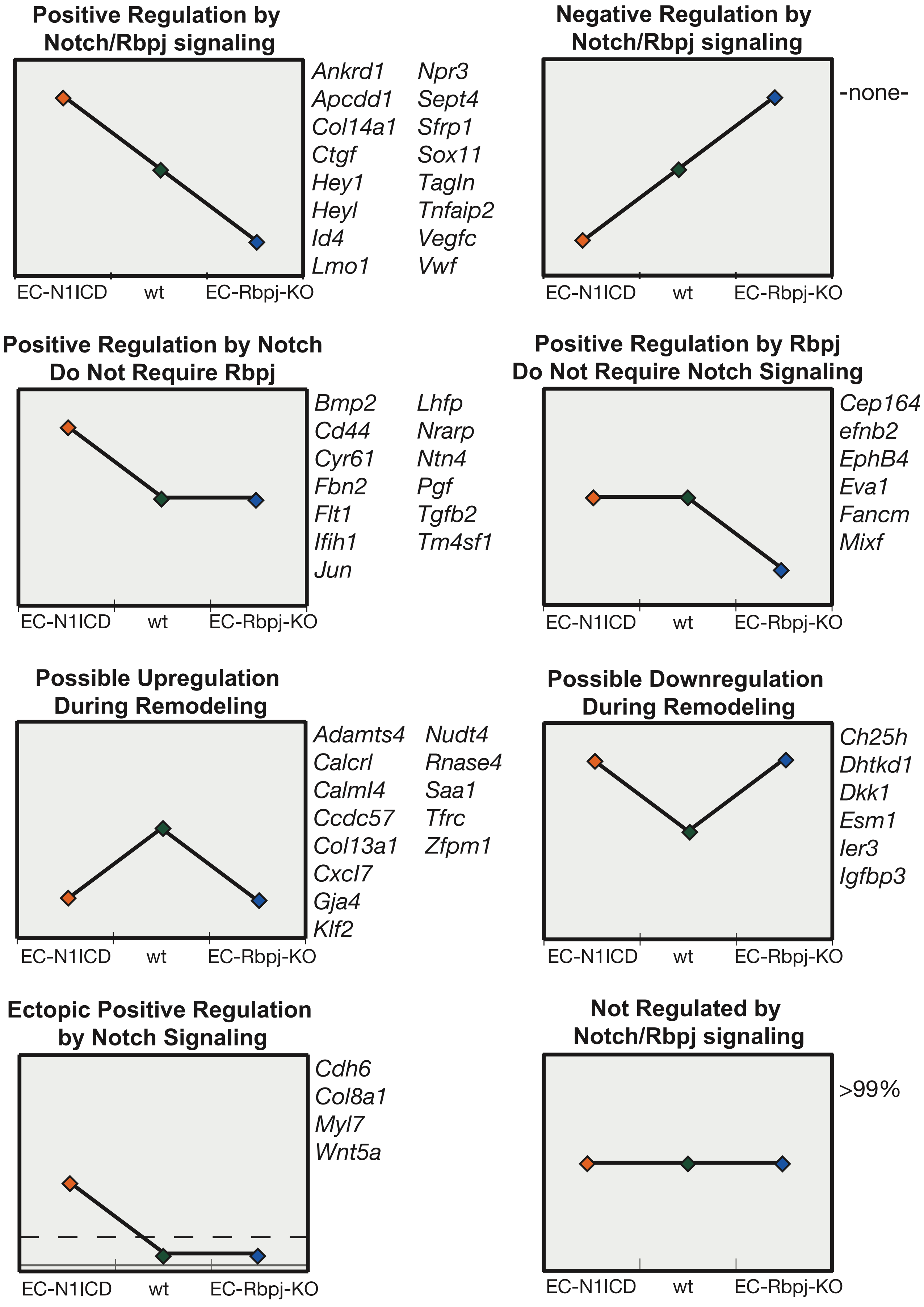

Positive Regulation by Notch/Rbpj signaling
Ankrd1
Apcdd1
Col14a1
Ctgf
Hey1
Heyl
Id4
Lmo1
Npr3
Sept4
Sfrp1
Sox11
Tagln
Tnfaip2
Vegfc
Vwf
EC-N1ICD
wt
EC-Rbpj-KO
Negative Regulation by Notch/Rbpj signaling
-none-
EC-N1ICD
wt
EC-Rbpj-KO
Positive Regulation by Notch Do Not Require Rbpj
Bmp2
Cd44
Cyr61
Fbn2
Flt1
Ifih1
Jun
Lhfp
Nrarp
Ntn4
Pgf
Tgfb2
Tm4sf1
EC-N1ICD
wt
EC-Rbpj-KO
Positive Regulation by Rbpj Do Not Require Notch Signaling
Cep164
efnb2
EphB4
Eva1
Fancm
Mixf
EC-N1ICD
wt
EC-Rbpj-KO
Possible Upregulation During Remodeling
Adamts4
Calcrl
Calml4
Ccdc57
Col13a1
Cxcl7
Gja4
Klf2
Nudt4
Rnase4
Saa1
Tfrc
Zfpm1
EC-N1ICD
wt
EC-Rbpj-KO
Possible Downregulation During Remodeling
Ch25h
Dhtkd1
Dkk1
Esm1
Ier3
Igfbp3
EC-N1ICD
wt
EC-Rbpj-KO
Ectopic Positive Regulation by Notch Signaling
Cdh6
Col8a1
Myl7
Wnt5a
EC-N1ICD
wt
EC-Rbpj-KO
Not Regulated by Notch/Rbpj signaling
>99%
EC-N1ICD
wt
EC-Rbpj-KO
