# Supplementary material for: Notch signaling regulates remodeling and vessel diameter in the extraembryonic yolk sac
Source: BMC Dev Biol. 2011 Feb 25;11:12. doi: 10.1186/1471-213X-11-12 (PMC3051915; doi:10.1186/1471-213X-11-12)
Supplement: Additional file 4 — Expression of genes encoding secreted factors in EC-N1ICD and EC-Rbpj-KO yolk sac tissues [file 1471-213X-11-12-S4.PDF]

Expression of genes encoding secreted factors in EC-N1ICD and EC-Rbpj-KO yolk sac tissues

Gene Symbol	Gene Name	Fold Change EC-N1ICD	Fold Change EC-Rbpj-KO
**Upregulated in EC-N1ICD**			
Bmp2	bone morphogenetic protein 2	2.35	1.11
Col14a1	collagen, type XIV, alpha 1	2.29	-1.96
Col8a1	collagen, type VIII, alpha 1	4.88	1.07
Ctgf	connective tissue growth factor	2.13	1.18
Cxcl12	chemokine (C-X-C motif) ligand 12	1.94	1.29
Cyr61	cysteine rich protein 61	2.63	1.66
Esm1	endothelial cell-specific molecule 1	3.41	3.51
Flt1	FMS-like tyrosine kinase 1	1.94	1.07
Ier3	immediate early response 3	2.01	2.10
Igfbp3	insulin-like growth factor binding protein 3	3.85	4.05
Ntn4	netrin 4	5.76	1.39
Rdh5	retinol dehydrogenase 5	2.06	1.39
Snn	stannin	1.97	1.63
Tgfb2	transforming growth factor, beta 2	3.11	1.32
Vegfc	vascular endothelial growth factor C	3.65	-2.50
Vtn	vitronectin	3.34	3.15
Vwf	Von Willebrand factor homolog	2.05	-3.33
Wnt4	wingless-related MMTV integration site 4	2.09	2.45
Wnt5a	wingless-related MMTV integration site 5A	2.47	-1.12
Pgf	placental growth factor	1.93	1.43
**Downregulated in EC-N1ICD**			
Adamts4	a disintegrin-like and metallopeptidase with thrombospondin type 1 motif, 4	-2.12	-2.44
Cxcl4	chemokine (C-X-C motif) ligand 2	-4.22	-1.11
Hhip	Hedgehog-interacting protein	-2.33	-1.39
Oit3	oncoprotein induced transcript 3	-3.70	1.53
Rnase4	ribonuclease, RNase A family 4	-2.14	-2.04
Saa1	serum amyloid A1	-2.39	-2.44
