# Supplementary material for: Notch signaling regulates remodeling and vessel diameter in the extraembryonic yolk sac
Source: BMC Dev Biol. 2011 Feb 25;11:12. doi: 10.1186/1471-213X-11-12 (PMC3051915; doi:10.1186/1471-213X-11-12)
Supplement: Additional file 7 — Primer pairs used for RT-PCR [file 1471-213X-11-12-S7.PDF]

Primer pairs used for RT-PCR

Gene	Forward Primer	Reverse Primer	Product (bp)
Hey1	TCCGCCACCATGAAGAGAGC	AACTTCGGCCAGGCATTCCC	627
Heyl	GAAACGGCGCAGAGACCGCATCAACA	TCCCAGGATGGCGAGCTGACTGTTCA	436
Notch1	TGCCTGTGCACACCATTCTGC	CAATCAGAGATGTTGGAATGC	247
Notch4	AAGCGACACGTACGAGTCTGG	ATAGTTGCCAGCTACTTGTGG	297
Dll4	AACTGTCCTTATGGCTTTGT	CACACTCGTTCCTCTCTTCT	520
Jag1	CCAGCCAGTGAAGACCAAGT	TCAGCAGAGGAACCAGGAAA	398
Flk1	GCCAATGAAGGGGAACTGAAGAC	TCTGGCTGCTGGTGATGCTGTC	538
Vegfa	CCTCCGAAACCATGAACTTTCTGCTC	CAGCCTGGCTCACCGCCTTGGCTT	593
Vegfb	GTCAAACAACTAGTGCCCAG	TGTCTGGGTTGAGCTCTAAG	447
Vegfc	CGTTCTCTGCCAGCAACATTAC	TGGCCTTTTCCAATACGATGG	570
Pgf	CAGCCAACATCACTATGCAG	GGGTGACGGTAATAAATACG	268
Tgfb2	GGAAAAAAACCAGTGGGAAGACCC	AAGCTGTTCGATCTTGGGCG	398
